# Dupilumab‐associated head and neck dermatitis: Rapid response with abrocitinib treatment

**DOI:** 10.1002/ski2.312

**Published:** 2023-12-13

**Authors:** Adinia Santosa, Yik Weng Yew

**Affiliations:** ^1^ National Skin Centre Singapore Singapore

## Abstract

Development or exacerbation of head and neck dermatitis (HN‐D) in association with dupilumab has been reported. Severity of HN‐D varies, and may persist even with discontinuation of dupilumab. Development or exacerbation of HN‐D is not yet completely understood, and various hypotheses have been made about the possible underlying pathophysiology. To date, there is no established treatment for HN‐D in association with dupilumab. We report 2 cases of HN‐D occurring following dupilumab treatment, with significant improvement of HN‐D following treatment with abrocitinib.

## 1 INTRODUCTION

Dupilumab has been proven to improve disease‐control in moderate‐to‐severe atopic dermatitis (AD). However, development or exacerbation of head and neck dermatitis (HN‐D) in association with dupilumab has been reported.^1^, ^2^ Severity of HN‐D varies, and may persist even with discontinuation of dupilumab.^1^ With approval of Janus Kinase inhibitors (JAK‐I) as treatment for AD, patients are presented with a new potential treatment option for refractory or difficult‐to‐treat areas.^3^ Herein, we report two patients with persistent HN‐D post‐dupilumab treatment, with subsequent improvement after abrocitinib, a selective JAK‐1 inhibitor.

## 2 CASE 1

**FIGURE 1:**
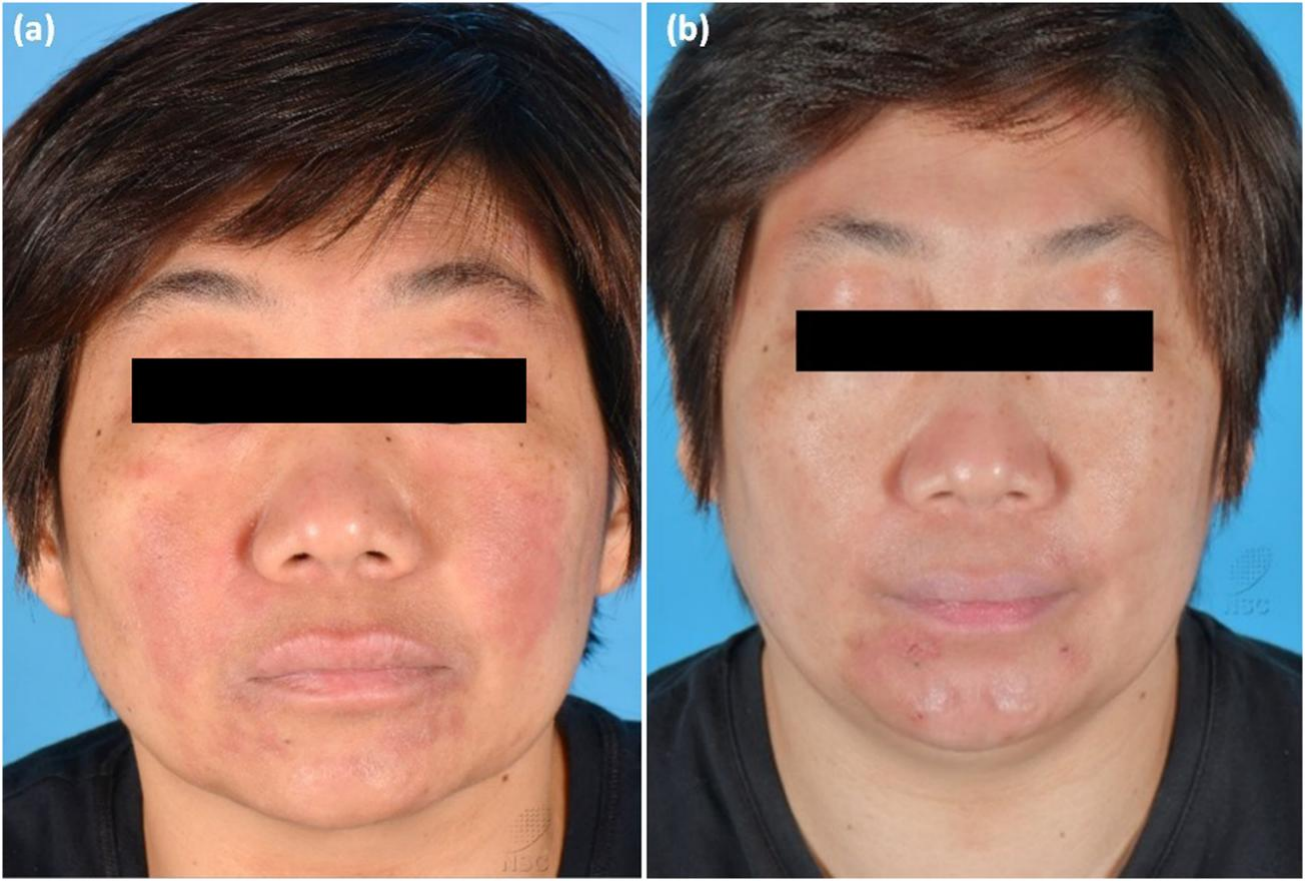
(a) Case 1 with HN‐D whilst on baricitinib, and (b) after 4 weeks treatment with abrocitinib.

A 51‐year‐old Chinese female patient with known AD since childhood has been on cyclosporine, azathioprine and methotrexate with unsatisfactory disease control. She was commenced on dupilumab with good overall improvement, but developed new, progressive erythematous patches and plaques on her face after 8 weeks. She denied any new contactants or medications. She was diagnosed with HN‐D and received mometasone 0.1% cream and protopic 0.1% ointment to the face whilst continued on dupilumab. HN‐D persisted after 6 months without any improvement. Brief courses of systemic prednisolone and itraconazole led to initial improvement, but HN‐D returned on cessation. As HN‐D persisted for 1 year after initiation of dupilumab, she was switched to baricitinib 4 mg daily. Initial mild improvement of HN‐D was seen within 2 months of commencing baricitinib, but she worsened thereafter. She was eventually started on abrocitinib 200 mg daily and experienced significant improvement of HN‐D within 4 weeks of treatment. HN‐D remains well controlled 6 months while on abrocitinib (Figure 1).

## 3 CASE 2

**FIGURE 2:**
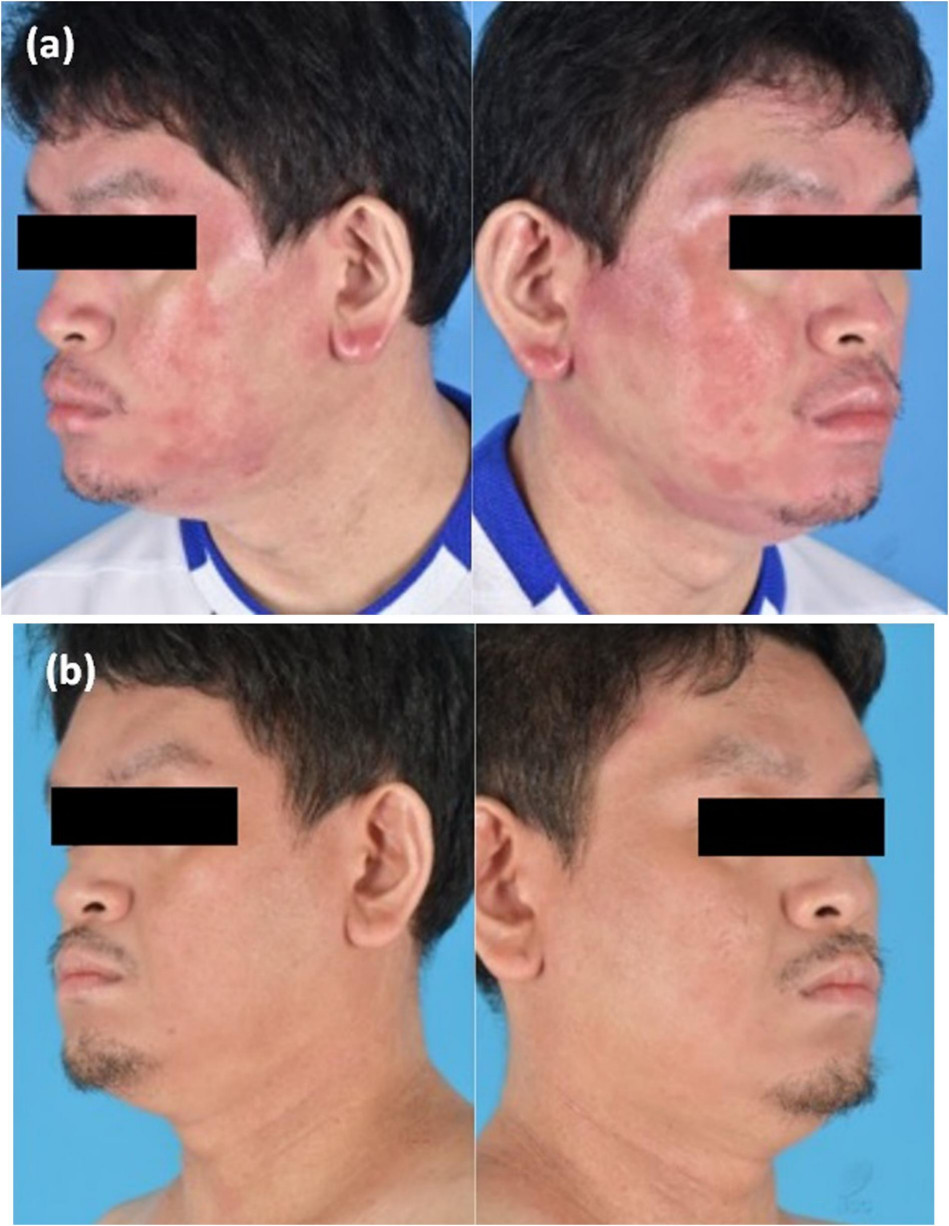
(a) Case 2 with HN‐D whilst on dupilumab, and (b) after 4 weeks treatment with abrocitinib.

A 41‐year‐old Chinese male patient with history of childhood eczema has received phototherapy, cyclosporine, methotrexate, azathioprine with limited disease control. He was started on dupilumab, but developed erythematous patches and plaques on cheeks, forehead, temples and submental regions within 3 months of treatment. Trial of topical miconazole did not lead to any improvement. Dupilumab was discontinued and he was switched to baricitinib 4 mg. While initial Improvement of HN‐D was observed within 2 weeks of treatment, he experienced another HN‐D flare within 6 weeks. He subsequently was switched to abrocitinib with good response to treatment within 4 weeks. Abrocitinib had to be tapered from 200 mg daily to 200 mg every other day in 4 months due to mildly raised transaminases, but overall eczema activity remained stable with significant improvement of HN‐D extent after 6 months (Figure 2).

## 4 DISCUSSION

In both cases, significant HN‐D developed after commencement of dupilumab, and improvement of HN‐D in our patients are likely attributed to both discontinuation of dupilumab as well as effect of abrocitinib.

Development or exacerbation of HN‐D is not yet completely understood, and various hypotheses have been made about the possible underlying pathophysiology.^1^ One hypothesis postulated that dupilumab activates the T helper 17 cell pathway, which was reported to be involved in the regulation of fungal colonisation and the promotion of Malassezia‐induced inflammation in a mouse model with atopy‐like skin.^4^ Antifungal treatment administered in patients with HN‐D symptoms was observed to lead to improvement of their symptoms.^5^ Both of our patients received anti‐fungal treatment, either as systemic itraconazole or topical miconazole, but only temporary and limited improvement was noted.

Baricitinib obtained approval from local health authorities for treatment of moderate to severe AD in November 2021, shortly followed by abrocitinib for the same indication a few months later. The persistence of HN‐D of our patients, even with the inclusion of adjunctive treatments, coincided with the approval of baricitinib. This circumstance led to the clinical decision to transition both patients to a treatment regimen involving baricitinib. Temporary initial improvement of HN‐D was observed in both patient within 6–8 weeks, but subsequently worsened. Both patients were eventually transitioned to abrocitinib, with good clinical response observed after 6 months.

Previous research has shown that JAK‐I can effectively manage head and neck eczema,^3^, ^6^ however, it is unclear whether such benefits extend to the context of HN‐D post‐dupilumab administration. Phase 3 JADE COMPARE study has revealed that abrocitinib can achieve an Eczema Area and Severity Index‐90 score in the head and neck region in a median time of approximately 2 months.^3^ Given the rapid improvement of HN‐D symptoms, abrocitinib may potentially be a treatment option in patients who experience new or exacerbation of HN‐D post‐dupilumab. Other JAK‐I as treatment of post‐dupilumab HN‐D symptoms have been reported for upadacitinib,^7^ a JAK‐1 inhibitor, and topical delgocitinib,^8^ a pan‐JAK‐I. There is emerging evidence on mechanism and efficacy of the different subclasses JAK‐I in this patient population. It has been postulated that JAK‐Is render broad suppression of cytokine‐mediated inflammation hence allowing improvement of HN‐D symptoms.^8^

These scenarios raise a point of consideration when choosing between dupilumab or the various subclasses of JAK‐inhibitors as AD treatment options. More studies are needed to evaluate the role of JAK‐inhibitors in dupilumab associated HN‐D.

## AUTHOR CONTRIBUTIONS

**Adinia Santosa**: Conceptualization (supporting); data curation (supporting); formal analysis (supporting); writing—original draft (lead); writing—review and editing (equal). **Yik Weng Yew**: Conceptualization (lead); formal analysis (lead); supervision (lead); writing—original draft (supporting); writing—review and editing (equal).

## CONFLICT OF INTEREST STATEMENT

None to declare.

## ETHICS STATEMENT

Not applicable.

## Data Availability

Data available on request due to patient privacy restrictions.
